# Action of Hyaluronic Acid as a Damage-Associated Molecular Pattern Molecule and Its Function on the Treatment of Temporomandibular Disorders

**DOI:** 10.3389/fpain.2022.852249

**Published:** 2022-03-18

**Authors:** Natália dos Reis Ferreira, Carolina Kaminski Sanz, Aline Raybolt, Cláudia Maria Pereira, Marcos Fabio DosSantos

**Affiliations:** ^1^Faculty of Medicine, Institute of Occlusion and Orofacial Pain, University of Coimbra, Coimbra, Portugal; ^2^Laboratório de Propriedades Mecânicas e Biologia Celular (PropBio), Departamento de Prótese e Materiais Dentários, Faculdade de Odontologia, Universidade Federal do Rio de Janeiro (UFRJ), Rio de Janeiro, Brazil; ^3^Programa de Engenharia Metalúrgica e de Materiais, COPPE, Universidade Federal do Rio de Janeiro (UFRJ), Rio de Janeiro, Brazil; ^4^Programa de Pós-Graduação em Neurociência Translacional, Instituto Nacional de Neurociência Translacional (INNT-UFRJ), Rio de Janeiro, Brazil; ^5^Programa de Pós-Graduação em Odontologia (PPGO), Universidade Federal do Rio de Janeiro (UFRJ), Rio de Janeiro, Brazil

**Keywords:** hyaluronic acid, damage-associated molecular patterns, synovial fluid, extracellular matrix, temporomadibular joint disorders

## Abstract

The temporomandibular joint is responsible for fundamental functions. However, mechanical overload or microtraumas can cause temporomandibular disorders (TMD). In addition to external factors, it is known that these conditions are involved in complex biological mechanisms, such as activation of the immune system, activation of the inflammatory process, and degradation of extracellular matrix (ECM) components. The ECM is a non-cellular three-dimensional macromolecular network; its most studied components is hyaluronic acid (HA). HA is naturally found in many tissues, and most of it has a high molecular weight. HA has attributed an essential role in the viscoelastic properties of the synovial fluid and other tissues. Additionally, it has been shown that HA molecules can contribute to other mechanisms in the processes of injury and healing. It has been speculated that the degradation product of high molecular weight HA in healthy tissues during injury, a low molecular weight HA, may act as damage-associated molecular patterns (DAMPs). DAMPs are multifunctional and structurally diverse molecules that play critical intracellular roles in the absence of injury or infection. However, after cellular damage or stress, these molecules promote the activation of the immune response. Fragments from the degradation of HA can also act as immune response activators. Low molecular weight HA would have the ability to act as a pro-inflammatory marker, promoting the activation and maturation of dendritic cells, the release of pro-inflammatory cytokines such as interleukin 1 beta (IL-1β), and tumor necrosis factor α (TNF-α). It also increases the expression of chemokines and cell proliferation. Many of the pro-inflammatory effects of low molecular weight HA are attributed to its interactions with the activation of toll-like receptors (TLRs 2 and 4). In contrast, the high molecular weight HA found in healthy tissues would act as an anti-inflammatory, inhibiting cell growth and differentiation, decreasing the production of inflammatory cytokines, and reducing phagocytosis by macrophages. These anti-inflammatory effects are mainly attributed to the interaction of high-weight HA with the CD44 receptor. In this study, we review the action of the HA as a DAMP and its functions on pain control, more specifically in orofacial origin (e.g., TMD).

## Background

Temporomandibular Disorders (TMD) have been widely described as a heterogeneous group of conditions that affect the Temporomandibular Joint (TMJ), the masticatory muscles, and associated structures. However, TMD is a highly generic term. In fact, it refers to a set of muscle (masticatory muscle disorders) and joint (intra-articular TMD) disorders (1). For example, anterior joint disc displacement and degenerative joint disease (DJD) are the most frequent intra-articular TMD. The pathophysiology of each of them is complex and encompasses multiple risk factors. Such factors, in turn, interact in different ways in each individual (2).

Furthermore, the chronicity of pain, with its peripheral and central mechanisms, such as peripheral and central sensitization, as well as the participation of psychogenic factors, make the complete understanding of its mechanisms especially challenging (3). Concerning the etiopathogenesis of articular TMD, those conditions were highly correlated to mechanical factors. However, the participation of more complex biological mechanisms such as immune system activation and inflammation activation has been acknowledged in recent years (4).

The mid-nineties have witnessed the introduction of the so-called Danger model. This term refers to a theory developed by Polly Matzinger to explain the activation of innate immunity (5). According to this model, a layer of cells and signals promotes the activation of innate immunity. Such activation would result from danger/alarm signals from injured cells such as those exposed to pathogens, toxins, mechanical injury, and others (5, 6). Those endogenous signals were further called alarmins or damage-associated molecular patterns (DAMPs). DAMPs are multifunctional and structurally diverse molecules that play important physiological intracellular roles in the absence of injury or infection (7).

Nonetheless, once released after cellular damage or stress, they promote the activation of the immune response (8). Most DAMPs are proteins and nucleotides derived from the cell nucleus or the cytoplasm, including the high mobility group 1 protein (HMGB1), heat shock proteins, histones, microRNAs, mitochondrial RNA, and ATP. However, not all DAMPs have an intracellular origin. Fragments from the degradation of the extracellular matrix (ECM) components such as fibrinogen, fibronectin, and hyaluronic acid (HA) might also activate the immune response under some circumstances (9). DAMPs can trigger and maintain immune responses in the absence of infectious agents, making them critical for the sterile inflammation seen in many diseases. Once released from damaged or inflamed tissues, DAMPs activate innate immune cells, including neutrophils, tissue macrophages, and dendritic cells through pattern recognition receptors (PRRs). Overall, DAMPs are endogenous molecules released as a response after cellular stress, damage, or other stimuli (7). Despite contributing to the hosts' defense, DAMPs trigger the immune system and promote an exacerbated and pathological response (7). DAMPs function as signaling mediators of stress responses and the immune response via specific membrane or intracellular receptors or after endocytic uptake. Remarkably, recent data suggest that PRPs are expressed by nociceptors, which may indicate that an increase in nociceptive activity is also part of the damage signals protection system. In addition, the presence of these PRPs in neurons, microglia, astrocytes, and oligodendrocytes has raised the hypothesis that DAMPs participate in chronic pain mechanisms (10).

## The Role of Hyaluronic Acid in Etiopathogenesis of Intra-Articular Temporomandibular Disorders

Intra-articular TMD consists of various joint disorders and diseases, such as disc displacements with or without reduction, DJD and subluxation. According to the taxonomy proposed by the Diagnostic Criteria for Temporomandibular Disorders, The terms osteoarthritis (OA), and osteoarthrosis are considered to denote subclasses of DJD (11). Many potential risk factors have been proposed associated with intra-articular TMD. Mechanical overload in the joint environment and microtrauma has been suggested as a critical piece in understanding the pathophysiology of DJD and TMJ disc displacements. Joint tissues are sensitive to physical stimuli. Hence, a dynamic mechanical load is an important stimulus to the mandibular growth and TMJ cartilage homeostasis (12). On the other hand, excessively aberrant mechanical loads on healthy joint tissues or physiologically normal loads on pathologically compromised cartilage, bones, disc, and joint ligaments lead to degeneration of joint tissues (13, 14). Parafunctional habits, especially awake bruxism, represent the main causes of TMJ overload and microtrauma. Multiple possible mechanisms might explain the role of joint overload in the pathogenesis of an intra-articular TMD (13).

One of the possible mechanisms associated with TMJ overload would be mediated by chondrocytes in the articular cartilage. According to this theory, chondrocytes present a response system to mechanical stimuli that promote an increase in metabolic activity as well as activation of the pathological process. In this process, the vascular endothelial growth factor (VEGF) is a fundamental component. VEGF induces chondrocyte apoptosis and regulates the release of matrix metalloproteinases (MMPs) and tissue inhibitors of metalloproteinases (TIMPs) within the articular cartilage (15). The reduction of TIMPs and the induction of MMPs result in an imbalance in the turnover of the components of the ECM that are degraded faster than formed (16). In addition to the damage to the articular cartilage, studies using the finite element model of the TMJ demonstrated that prolonged clenching could cause damage to the articular disc due to a substantial increase in shear stresses in this structure (16, 17).

Another mechanism associated with overload is the reduction in joint lubrication capacity. Essentially, there are two types of lubrication in synovial joints: boundary lubrication and fluid film lubrication (18). According to fluid film lubrication mechanisms, friction reduction occurs due to a thin film formed by synovial fluid separating the joint surfaces. This type of lubrication is directly related to the rheological characteristics of synovial fluid, such as its viscosity and elasticity (19, 20). Thus, HA is essential in this lubrication mechanism since it represents a vital component for determining the synovial fluid rheological properties, especially viscoelasticity. This property is strongly related to the HA molecular weight and, to a lesser extent, to its concentration (18). In boundary lubrication, the joint surfaces are protected by the layer of lubricants absorbed by them, thus avoiding surface-to-surface contact and reducing friction (20). The lubricating capacity in this mechanism is related to the chemical properties of the lubricant, and the synovial fluid viscosity plays a less relevant role. Thus, the main molecules involved in boundary lubrication are surface-active phospholipids and lubricin (13).

**Figure 1:**
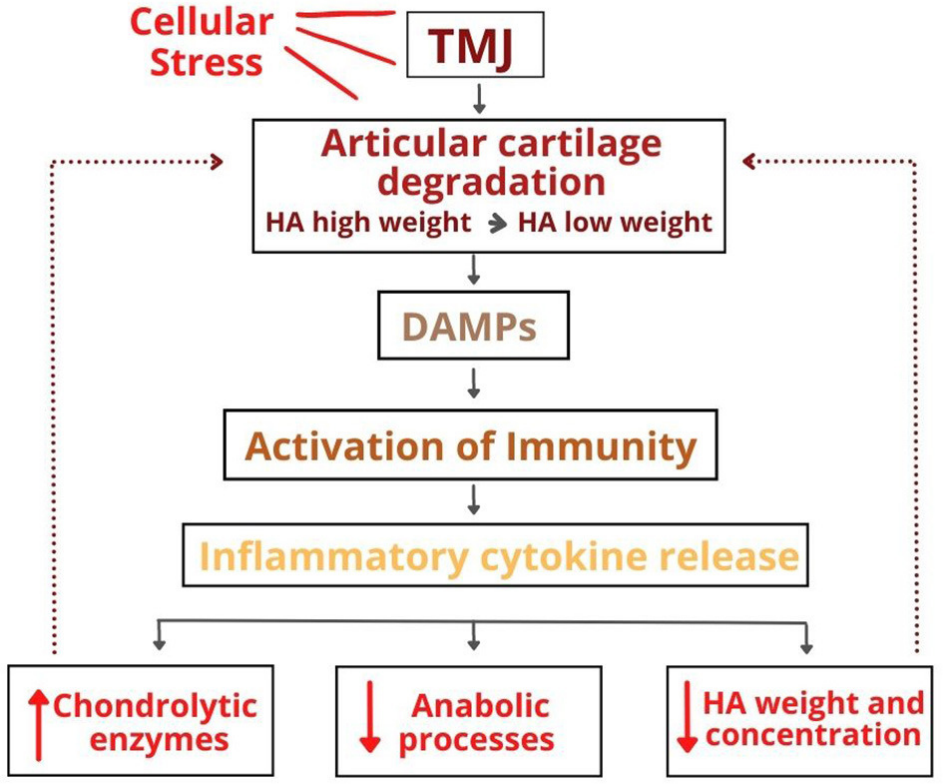
The role of hyaluronic acid in the etiopathogenesis of temporomandibular disorders.

In a prolonged period of overload, the tinny film of the synovial fluid that prevents a direct contact between the joint surfaces does not work, leaving only the boundary lubrication mechanism to protect the joint components. However, overload also acts on the composition of synovial fluid through oxidative stress. According to this mechanism, the increased production of reactive oxygen species, both through hypoxia-reperfusion mechanisms and the presence of inflammatory cytokines, lead to oxidative stress due to a disturbance in the balance between the production of reactive oxygen species and the antioxidant defense (21). The oxidative stress can act in different ways on joint tissues, such as damaging different molecules of the articular cartilage, modulating pro-inflammatory cytokines, and degrading synovial fluid HA (15, 19). In addition to promoting the degradation of high-molecular-weight HA in the synovial fluid, oxidative stress also inhibits HA synthesis by synoviocytes, affecting the viscoelastic property of the synovial fluid. The Figure 1 illustrates this whole process.

Furthermore, the reduction of synovial fluid HA allows the degradation of surface-active phospholipids since the adhesion of these molecules to the HA protects them from degradation by phospholipase A2. Thus, HA fragmentation will affect joint lubrication mechanisms, increasing friction between joint surfaces (13). In fact, the study conducted by Asakawa-Tanne et al. (22) evaluated the coefficient of friction in the TMJ after injection of hyaluronidase into the synovial fluid in an animal model. After treatment with this enzyme, the results showed a significant increase in the coefficient of friction and gene expression of IL-1β, COX-2, and MMPs 1, 3, and 9. The authors of that study concluded that compromised TMJ lubrication is associated with an increase in friction and wear on the cartilage surface of the mandible condyle. These events are accompanied by the release of pro-inflammatory mediators and ECM degradation (22).

OA was considered a wear and tear disease for many years due to the undeniable role of mechanical factors in its pathogenesis. However, recent evidence has shown the participation of inflammation in the degeneration of joint components (23). The relationship between overload and inflammation in the pathogenesis of OA has not yet been clarified. However, some authors suggest that the changes caused by a mechanical overload may be responsible for triggering a complex cell signaling process that would culminate in the inflammatory process (15). Inflammation in OA is mediated by innate immunity, activated by small endogenous molecules called alarmins or DAMPs (8, 9).

Disc displacement has also been identified as a risk factor for the development of DJD since some observational studies demonstrate a significant association between disc displacement without reduction and DJD (24, 25). However, other theories postulate that DJD precedes TMJ disc displacements. Some authors suggest that irregularities on the articular surface would lead to an incompatibility between the disc and the condylar surface. In comparison, other authors suggest that the inflammatory environment found in DJD would be responsible for the degradation of HA, with a consequent reduction in lubrication and increased friction that would culminate in disc displacement (13, 19).

## Hyaluronic Acid as A Damp

The ECM is a type of scaffold profusely found in all organs and tissues. It is responsible not only for adequate cellular support but also contributes to individual and collaborative cellular functions at the organ level. The ECM has an important feature: functional diversity. The ECM is made up of different molecules, elements, and structures, including proteoglycans, hyaluronans, collagen, fibronectins, laminins, and glycosaminoglycans (26). The various components of the ECM form an interconnected network that contains many connections between cells and their adhesion receptors. The composition and organization of the ECM contribute to each tissue's unique physical or biomechanical properties. Cell surface receptors are responsible for signal transduction in ECM cells, which regulate growth, migration, morphogenesis, and cell differentiation. The ECM of each tissue has its particularity and architecture. For instance, the TMJ ECM is predominantly composed of collagen fibers, proteoglycans, glycosaminoglycans, such as HA of high molecular weight, water, growth factors, cytokines, and other important molecules that play the roles of support and regulation of the anatomic structure, in this example, the TMJ. Among all those components that constitute the EMC of the TMJ, the HA is one of the most studied (27, 28).

HA belongs to the family of glycosaminoglycans (GAGs) naturally found in many tissues, including connective tissue, cartilage, and synovial fluid. It is a long-chain linear polysaccharide formed by repeating units of the disaccharide D-glucuronic acid and N-acetyl-D-glucosamine, forming chains with up to 25,000 units (29). As a result of this large number of disaccharide units, HA molecules have extremely high molecular weights that can reach up to 10,000 kDa. In the healthy synovial fluid, HA is abundant, and most of it has a high molecular weight of around 6,000 kDa. Nevertheless, it is possible to find HA with a molecular weight of <500 kDa (18). In addition, it plays a crucial role in tissue homeostasis and integrity, acting as a support and maintaining multivalent extracellular interactions between ECM molecules (26). In degenerative joint diseases, the decomposition of the ECM is an essential mechanism in the pathological process (15). However, the role of ECM and HA has been better explored, for example, *in vivo* studies have shown that low molecular weight HA possibly regulates inflammation through the release of inflammatory cytokines and potentiation of the immune response, and it is associated with attenuated hyperalgesia, which can be prevented with high molecular weight HA. This inflammatory modulation is attributed to the interaction of HA with the CD44 receptor and would be directly related to its molecular weight (30). The HA contents of ECMs and pericellular matrices are regulated by hyaluronidases, which are responsible for degrading HA in fragments with different functions. It is suggested that these degradation products may act as DAMPs (9).

DAMPs are multifunctional and structurally diverse molecules that play important intracellular roles in the absence of injury or infection. However, once released after cellular damage or stress, these molecules promote the activation of the immune response (8). Most DAMPs are proteins and nucleotides from the cell nucleus or cytoplasm, such as high mobility group 1 protein, heat shock proteins, histones, microRNA, mitochondrial RNA, and ATP. However, not all DAMPs are of intracellular origin. Fragments from the degradation of ECM components, such as HA, fibrinogen, and fibronectin, can also act as activators of the immune response (9).

High-molecular-weight HA is catabolized in HA of lower molecular weight during tissue injury. This can result from endogenous and/or microbial hyaluronidases (in case of infection), mechanical forces, or oxidation. The low-molecular-weight HA would have the ability to act as a pro-inflammatory marker, promoting activation and maturation of dendritic cells, the release of pro-inflammatory cytokines such as interleukin 1 beta (IL-1β), tumor necrosis factor α (TNF-α), IL-6, and IL-12, by various types of cells. Low-molecular-weight HA also increases the expression of chemokines and promotes cell proliferation. Many of the pro-inflammatory effects of low-molecular-weight HA are attributed to its interactions with toll-like receptors (TLR 2 and TLR 4). On the other hand, high-molecular-weight HA found in healthy tissues would produce an anti-inflammatory effect by inhibiting cell growth and differentiation, decreasing the production of inflammatory cytokines, and reducing phagocytosis by macrophages (31). Such anti-inflammatory effects of high molecular could be putatively attributed to its interactions with the CD44 receptor.

The enzymatic degradation of the cartilaginous tissue promoted by MMPs due to a mechanical overload results in abundant molecules production and ECM fragments that act as DAMPs in the joint environment. When released into the extracellular environment, these molecules bind to pathogen recognition receptors, such as TLRs. TLR2 and TLR4 are capable of binding to many ECM fragments that arise from the degraded cartilage. These fragments include low-molecular-weight HA, tenascin C, fibronectin, and aggrecan (8, 32). In addition to ECM fragments, other DAMPs generated by cellular stress have been found in the synovial fluid of patients with DJD, including high-mobility box 1 protein, uric acid, ATP, thymosin β4, and several S100 proteins. The binding between TLRs and DAMPs results in the activation of inflammatory signaling pathways, including the nuclear factor-κB (NF-κB), mitogen-activated protein kinase (MAPK), and type I interferon pathways, with subsequent release of cytokines, chemokines, and proteases (33). As a result, there is inflammation and degradation of the articular cartilage, resulting in the release of new DAMPs, thus establishing a vicious cycle (8).

Several types of joint cells respond to DAMPs. Notwithstanding most of the innate immune activation and cytokine production in OA is attributed to the action of synovial macrophages, there is also a direct, contributory role for other joint cells, including synoviocytes, fibroblasts, and chondrocytes. Pain is a protective and an alarm signal capable of activating neuroplastic mechanisms such as learning and memory which protect an injured region through behavioral strategies to avoid further damage. With this respect, it is expected that DAMPs, in addition to their role in the innate immunity, also contribute to the induction and maintenance of nociceptive impulses (10). There is scientific evidence that DAMPs act directly or indirectly in nociceptors activation. Two studies conducted by Ferrari et al. (34, 35) demonstrated that the injection of HA of low molecular weight and hyaluronidase causes hyperalgesia in experimental models of antihyperalgesia induced by high molecular weight hyaluronan (HMWH). They also found that both peptidergic (IB4 neurons) and non-peptidergic (IB4) neurons are involved in LMWH-induced sensitization, and that this effect is mediated by the CD44 receptor (34, 35).

Another relevant issue in TMDs is the high prevalence in females, with a peak incidence in young adults. The low prevalence of TMD in childhood and in the postmenopausal period suggests the participation of sex hormones, such as estrogen, in the pathophysiology of this disorder (36). Bonet et al. (37) demonstrated that the intradermal administration of low molecular weight HA induces a mechanical hyperalgesia of similar magnitude in male and female rats. However, the duration of hyperalgesia is longer in females. The difference in the duration of the hyperalgesia between male/female rats was eliminated by bilateral ovariectomy, indicating this effect highly depends on the estrogen levels (37).

## Basis of Inflammation in TMD

Inflammation is highly associated with TMJ pain found in many types of TMD, and this is part of the complex mechanism of pain modulation (36, 38). Previous studies demonstrated that patients diagnosed with TMD have higher levels of cytokines in the synovial fluid of the TMJ compared to healthy controls. The main differences were related to the following cytokines: granulocyte-macrophage colony-stimulating factor (GM-CSF), interferon-gamma (IFN-γ), IL-1β, IL-2, IL-6, IL-8, IL-10, and TNF-α. IL-1β followed by IL-6 and GM-CSF were the cytokines that most differentiated both groups (39). In fact, there is scientific evidence that the pain sensitivity reported by TMD patients occurs in the presence of a systemic hyperinflammatory state. A case-control study of 344 females observed changes in circulating cytokine levels in patients with TMD with and without widespread palpation tenderness (WPT). A distinct profile of cytokines was observed in females with localized and widespread manifestations of chronic pain compared to pain-free controls. Significant higher levels of the pro-inflammatory cytokine IL-8 were found in TMD+WPT individuals. On the other hand, the pro-inflammatory cytokine MCP-1 and the anti-inflammatory cytokine IL-1ra were increased in TMD–WPT cases compared to controls (40). A systemic hyperinflammatory phenotype observed in women with TMD was confirmed in another study that reported a positive correlation between the levels of IL-6 in monocytic cells isolated from the peripheral blood of research volunteers and their self-reported pain on the Visual Analogue Scale (VAS) (36).

Unstimulated monocytes from TMD patients expressed eight times the amount of IL-6 compared to the unstimulated monocytes cells from the control group. The stratification of TMD group according to the VAS in low self-reported pain group (VAS < 4.5) and high self-reported pain group (VAS > 4.5) was correlated to the monocytic TLR4 responsiveness. The study showed increased levels of IL-6 following the stimulation with TLR4 ligand alone and in combination with estrogen in monocytes from the patients presented higher VAS scores, demonstrating the presence of a TLR4-monocytic hyperresponsive phenotype in women with TMD (36). Alterations in immune response associated with systemic inflammation could be related to longer pain periods and higher pain magnitude taking to poorer clinical function in TDM, as demonstrated in a study that evaluated the levels of cytokines, chemokines, autoantibodies compared to pain severity in female TMD and clinical indices of TMD. According to the findings of that study, increased levels of IL-8 and IgG were observed in a high pain disability group. Other inflammatory markers such as IL-2, -8, -13, IFN-γ, RANTES, PGE2, and thrombopoietin significantly affect indices reflecting jaw function, generalized pain intensity, and health-related quality of life (41). Put together, these results indicate the importance of an ongoing inflammatory process in patients with TMD. This process is regulated by several factors. Among them, epigenetics plays a crucial role and has been explored in several works.

## Additional Molecular Mechanisms Putatievely Involved in TMD

Epigenetics refers to the study of heritable changes in gene expression without changing the underlying DNA sequence (42). The search for comprehension of the pathophysiology related to the development of pain in TMD has highlighted the role of the epigenetic mechanisms in this process. The epigenetic mechanisms include DNA methylation, histone modifications, and the expression of microRNAs (miRNAs) (43). Acetylation and deacetylation of histones represent one of the most important epigenetic mechanisms. They regulate the condensation of chromatin, allowing, or preventing the process of gene transcription. Both *in vivo* and *in vitro* studies have associated the expression of L-1β to the histone deacetylase HDAC10 in synovial specimens from patients suffering from DJD. The knockdown of HDAC10 prevents the inflammatory activation of synovium-derived mesenchymal stem cells mediated by IL-1β (44). In fact, it has been demonstrated that IL-1β acts as a potent mechanical and thermal hyperalgesic agent (45) and exerts a role in the degradation and the inflammatory process underlying TMD (46).

DNA methylation array analysis demonstrated that in distinct stages of DJD could be distinguished from the healthy mandibular head cartilage by their distinct patterns of DNA methylation. Moreover, region of interest (ROI) analysis demonstrated 9,489 differentially methylated regions in samples of osteoarthritic TMJ cartilages compared to control samples. Roughly 76% of these were in the promoter regions, and the number of hypomethylated regions was higher than the number of hypermethylated regions. Genes associated to inflammation or immune processes, such as TNF-α, TNFRSF1A, TRAF2, IL-7, IL2RA, IL4RA, IL9RA, and IL27RA, demonstrated a significant decrease of DNA methylation in cartilages of DJD. The loss of methylation and the consequent activation of these inflammatory genes may trigger a signaling cascade leading to an intense inflammatory response in DJD (47).

MicroRNAs (miRNAs) have been considered pain biomarker. These molecules are associated with the prediction of responsiveness to analgesic drugs and individual risks to the development of chronic neuropathic pain (48). miRNAs are a class of small, non-coding molecules interacting with the 3′ untranslated region (UTR) of multiple target miRNAs that play a crucial role in gene regulation. The participation of miRNAs in pain processing has been demonstrated in a wide range of experimental pain models and clinical pain disorders. Nonetheless, there is a lack of robust data describing profiles of miRNAs in TMD (49). A miRNA microarray analysis performed in synovial fibroblasts isolated from the TMJ patients with DJD showed that 14 miRNAs are differentially expressed in DJD patients compared to normal synovial fibroblasts. Notably, miR-221-3p displayed the most significant difference (five-fold lower) in the synovial fibroblasts of patients with DJD, compared to the control group. *In silico* analysis showed that 3′-UTR of Ets-1, a member of the family of Ets transcription factors and associated with regulation of immune cell function (50), was complementary to miR-221-3p seed sequence. Using a dual-luciferase reporter assay, the authors demonstrated that the miR-221-3p suppressed the Ets-1 expression by directly binding to its 3′-UTR and its downstream molecules, MMP1 and MMP9 (51). In addition, circulating miRNAs expression profiles for the neuropathic pain demonstrated a downregulation of miR-221 in whole blood samples obtained from rats submitted to spinal nerve ligation (SNL) surgery (52). However, the relationship between miR-221 and neuropathic pain and neuroinflammation still is not well-established. One study demonstrated that inhibition of miR-221 caused a decrease in neuroinflammation and neuropathic pain. Treatment with the miR-221 inhibitor significantly induced an increased expression of the suppressor of cytokine signaling 1 (SOCS1), a direct target of miR-221, and inhibited the NF-κB and p38 MAPK signaling pathways (51). Other miRNAs such as miR-101a-3p (53) and miR-21-5p (54) have been correlated to the DJD. For example, one study found that the miR-101a-3p was decreased in an inflammatory model of DJD (53), and its neuropathic pain-attenuating activity was demonstrated in chronic constriction injury rat models through targeting mTOR (55). On the other hand, the upregulation of miR-21-5p was associated with cartilage matrix degradation and progression of the DJD (54). Another study revealed that miR-21 increases neuronal excitability via TLR8, and that this miRNA was involved in the maintenance of neuropathic pain (56).

## Viscossuplementation With HA as a Treatment of TMD

Viscosupplementation, by intra-articular injection of HA, has been used globally for the clinical management OA in symptomatic patients. At the very beginning, it was believed that the mechanisms of action of the HA were purely mechanical. This concept supported the therapeutic use of HA in the reestablishment of joint lubrication (57). With the implementation of HA injections and the performance of numerous clinical trials investigating its effectiveness, it was found that HA provided prolonged relief in joint pain, leading to questions regarding its purely mechanical mechanisms of action (58).

The mechanisms of action of HA injections in the DJD are not fully understood. However, it has been speculated that HA acts in several molecular signaling pathways and different types of cells within the joint environment. Therefore, it would contribute to the homeostasis of synovial joints. As already mentioned, endogenous HA plays a fundamental role in danger/injury and tissue repair signaling. Thus, it is expected that exogenous HA will also participate in these complex mechanisms (59). It has been speculated that intra-articular HA injections have anti-inflammatory and analgesic activity and promote the protection of joint tissues. Its action results from the interaction of HA with several cell receptors, such as the CD44 receptor, the intercellular adhesion molecule −1, and the hyaluronan-mediated motility receptor (58, 60, 61).

The anti-inflammatory activity produced by HA occurs mainly because of its binding to the CD44 receptor. According to some authors, HA is responsible for reducing the expression of IL-1β and TNF-α. The inhibition of the expression of these OA dominant catabolic cytokines would result in an overall reduction in the expression of the pro-inflammatory molecules (60). A clinical study carried out recently by Jia et al. (62), found a significant reduction in the concentration of IL-1β and IL-18 after an injection of HA in an individual with DJD (62). The randomized clinical trial conducted by Tang et al. (63) also found a significant reduction in the main components of the plasminogen activator system in the synovial fluid of DJD patients after intra-articular injections of HA (63). The suppression of IL-1β leads to a decline in the activation of MMPs 1, 2, 3, 9, and 13, which decreases the degradation of the ECM of the articular cartilage and promotes the apoptosis of chondrocytes. The anti-inflammatory effects are further reinforced by the reduced expression of other pro-inflammatory mediators, such as IL-6, IL-8 prostaglandin E2, free radicals, and increased anti-inflammatory cytokines (58).

In addition to the decrease in the catabolic activity promoted by the reduction in the expression of inflammatory cytokines, it is assumed that HA promotes the synthesis of ECM molecules (61). In addition, HA binding to the hyaluronan-mediated motility receptor would promote tissue repair and regulation of cellular responses to growth factors (60). Exogenous HA also contributes to joint protection through its viscoinduction capacity, in other words, its ability to restore average HA production by synovial fibroblasts (64). HA would be considered a disease-modifying drug (58, 64). It has been also attributed to exogenous HA an analgesic activity. Exogenous HA would act by reducing the levels of inflammatory cytokines that promote the activation of nociceptors and the direct action of HA on nociceptors. In fact, experimental studies have shown that HA of high molecular weight reduces the hyperalgesia associated with inflammatory pain (35, 37). For example, the studies conducted by Ferrari et al. demonstrated that high molecular weight HA attenuates and prevents the hyperalgesia caused by low molecular weight HA, hyaluronidase and inflammatory mediators (34, 35). According to these articles, the analgesic effect of high molecular weight HA is substantially related to its effects on nociceptor function through the signaling of second messengers involved in nociception (34, 35). For instance, it has been demonstrated that the inhibitors of CD44 second messengers such as RhoA (a member of the Rho family of GTPases), phospholipase C, and phosphatidylinositol (PI) 3-kinase gamma (PI3Kγ) mediate the HMWH-induced antihyperalgesia in rats with oxaliplatin and paclitaxel chemotherapy-induced peripheral neuropathy (CIPN) (65). In addition, phosphoinositide 3-kinase (PI3K)γ and protein kinase B (AKT) are also important to HMWH-induced anti-prostaglandin E2 (PGE2) anti-hyperalgesia (66). Noteworthy the differences in the experimental models used in each study (e.g., CIPN and inflammatory pain) must be considered. For example, one study showed that a decrease in the expression of TACAN, a mechanotransducing ion channel in nociceptors, reduces the hyperalgesia produced by inflammatory mediators but not changes the effects of chemotherapeutic agents (67). Interestingly, Bonet et al. (68) found differences in the analgesic effect between female and male rats. The authors demonstrated that high molecular weight HA-associated antihyperalgesia is mediated by CD44 and TLR 4 receptors in male rats, while in female rats the TLR 4 receptor does not participate in this process (68). A dimorphic involvement of TLR4 in HMWH-induced anti-hyperalgesia, that is sex hormone-dependent has been also demonstrated in another study (69). Caires et al. (70) suggest that HA has the ability to modulate the polymodal transient receptor potential vanilloid subtype 1 (TRPV1). According to these authors, HA decreases TRPV1 frequency opening. Therefore, it reduces the excitability of peripheral nociceptive neurons and lower their responsiveness to noxious stimuli (70). However, the assessment of these mechanisms is based mainly on *in vitro* or animal model studies. Therefore, robust clinical studies to are still needed to confirm such findings.

## Conclusion

DAMPs are multifunctional and structurally diverse molecules that activate an immune response when released after cellular injury or stress. Most DAMPs are proteins and nucleotides derived from the cell nucleus or cytoplasm, including histones, microRNA, mitochondrial RNA, and ATP. Nonetheless, not all DAMPs have an intracellular origin. For example, fragments from MEC may also play a role in the immune response. HA is one of those products. In the scenario of tissue injury, the high-molecular-weight HA that constitutes the ECM of several tissues is catabolized. This process results in a HA of lower molecular weight, which acts as a pro-inflammatory marker, promoting the activation and maturation of immune cells and the release of pro-inflammatory cytokines. In addition, it increases the expression of chemokines and stimulates cell proliferation. Many of the pro-inflammatory effects of low-molecular-weight HA are attributed to its interactions with the activation of TLRs, especially TLR 2 and TLR 4. Conversely, the high-molecular-weight HA present in healthy tissues exerts an opposite effect. High weigh HA has anti-inflammatory properties. It inhibits cell growth and differentiation, reduces the production of inflammatory cytokines, and reduces phagocytosis. Such effects are often linked to the interaction of high-weight HA with the CD44 receptor. In this study paper, we provide a comprehensive review of the activity of HA as DAMP, particularly in the context of inflammatory TMD.

## Author Contributions

NF, CS, and CP conceived the study, collected the data, analyzed the data, and led the writing. AR and MD analyzed the data and drafted the manuscript. All authors contributed to the article and approved the submitted version.

## Conflict of Interest

The authors declare that the research was conducted in the absence of any commercial or financial relationships that could be construed as a potential conflict of interest.

## Publisher's Note

All claims expressed in this article are solely those of the authors and do not necessarily represent those of their affiliated organizations, or those of the publisher, the editors and the reviewers. Any product that may be evaluated in this article, or claim that may be made by its manufacturer, is not guaranteed or endorsed by the publisher.
